# Oxidative stress and S-100B protein in children with bacterial meningitis

**DOI:** 10.1186/1471-2377-9-51

**Published:** 2009-10-08

**Authors:** Sherifa A Hamed, Enas A Hamed, Madeha M Zakary

**Affiliations:** 1 Department of Neurology, Faculty of Medicine, Assiut University, Assiut, Egypt; 2 Department of Physiology, Faculty of Medicine, Assiut University, Assiut, Egypt; 3 Department of Biochemistry, Faculty of Medicine, Assiut University, Assiut, Egypt

## Abstract

**Background:**

Bacterial meningitis is often associated with cerebral compromise which may be responsible for neurological sequelae in nearly half of the survivors. Little is known about the mechanisms of CNS involvement in bacterial meningitis. Several studies have provided substantial evidence for the key role of nitric oxide (NO) and reactive oxygen species in the complex pathophysiology of bacterial meningitis.

**Methods:**

In the present study, serum and CSF levels of NO, lipid peroxide (LPO) (mediators for oxidative stress and lipid peroxidation); total thiol, superoxide dismutase (SOD) (antioxidant mediators) and S-100B protein (mediator of astrocytes activation and injury), were investigated in children with bacterial meningitis (n = 40). Albumin ratio (CSF/serum) is a marker of blood-CSF barriers integrity, while mediator index (mediator ratio/albumin ratio) is indicative of intrathecal synthesis.

**Results:**

Compared to normal children (n = 20), patients had lower serum albumin but higher NO, LPO, total thiol, SOD and S-100B. The ratios and indices of NO and LPO indicate blood-CSF barriers dysfunction, while the ratio of S-100B indicates intrathecal synthesis. Changes were marked among patients with positive culture and those with neurological complications. Positive correlation was found between NO index with CSF WBCs (r = 0.319, p < 0.05); CSF-LPO with CSF-protein (r = 0.423, p < 0.01); total thiol with LPO indices (r = 0.725, p < 0.0001); S-100B and Pediatric Glasow Coma Scores (0.608, p < 0.0001); CSF-LPO with CSF-S-100B (r = 0.482, p < 0.002); serum-total thiol with serum S-100B (r = 0.423, p < 0.01).

**Conclusion:**

This study suggests that loss of integrity of brain-CSF barriers, oxidative stress and S-100B may contribute to the severity and neurological complications of bacterial meningitis.

## Background

Bacterial meningitis is the most severe and frequent infection of the central nervous system (CNS). It remains an important public health problem worldwide[1]. Even with antimicrobial therapy and the availability of advanced intensive care, the mortality rate of bacterial meningitis is ~25% in industrialized countries [2] and much higher in the developing world[1]. Several studies confirmed that brain damage is responsible for neurological sequelae in 50% of survivors after bacterial meningitis. Many of the complications may persist to adulthood[3,4]. Bedford et al. [3] evaluated 1584 children aged 5 years who had survived bacterial meningitis during the first year of life and found a 10-fold increase in the risk of having moderate to severe disabilities with 7.5% had learning difficulties, 8.1% had neuro-motor disabilities, 7.3% had seizure disorders, 25.8% had hearing problems and 11.9% had behavioral problems. Grimwood et al. [4] evaluated 109 children at adolescence (12 years of age) who had survived bacterial meningitis at 7 years of age and found that many of the deficits identified at the 7-year follow-up had persisted, with 9% of the patients had major neurological, auditory, or intellectual impairments and 30% had less severe disabilities, compared with 11% of the controls.

The mechanisms of CNS damage during meningitis have not been conclusively identified. During bacterial infections, neutrophils and macrophages gather at the site of infection to combat the microorganisms. Blood-derived and brain-resident immune cells employ reactive oxygen species (ROS) as part of their host defense mechanisms against invading bacteria. In this process, these cells consume molecular oxygen, which is converted into toxic superoxide anion (O^2.^), hydrogen peroxide and hydroxyl radical [5-7] Under normal circumstances, ROS are eliminated by cellular enzymatic [as superoxide dismutase (SOD) and catalase] and non-enzymatic [as glutathione and uric acid] antioxidants defenses[8]. Thus, if ROS are not effectively removed due to exhaustion of antioxidant capacity, an imbalance between oxidant and antioxidant activities will result in oxidative stress which includes peroxidation of membrane lipids, as well as damage on proteins and DNA. This may contributes to the development of several pathologic processes in bacterial meningitis[8,9]. On the other hand, astrocytes are the most abundant cell type in the brain and involved in a variety of important activities for the nervous system, including a protective role against damage induced by ROS[10]. S-100B is a calcium binding protein physiologically produced mainly by astrocytes in CNS and has been implicated in the development and maintenance of the nervous system. Although the mechanism of S-100B secretion is unknown, it appears to be affected by oxidative stress. At nanomolar concentrations (normal levels), S-100B is able to protect neurons against glutamate toxicity[11]. On the other hand, high levels of S-100B (micromolar) as a result of glial damage or astrocytic reactions to neural injury (reactive astrogliosis) will further increase neuronal damage[12]. High levels of S-100B have been considered as a biomarker that could indicate damage or dysfunction of CNS[13].

The relationship between S-100B and oxidative stress/antioxidant mechanisms in patients with bacterial meningitis is barely determined. Given the fact that improvements in antibiotic therapy are unlikely to substantially change the outcome of meningitis, it is imperative to continue efforts to obtain a better understanding of the pathogenesis of brain damage resulting from meningitis.

### Aim of the work

The present study may contribute to further understanding of factors involved in brain compromise associated with bacterial meningitis. Oxidant and antioxidant activities were assessed by measuring serum and CSF levels of NO, lipid peroxide (LPO) (markers of oxidative stress and lipid peroxidation); total Thiol and superoxide dismutase (SOD) (markers of antioxidant activity). S-100B protein is a marker of astrocytic reaction or glial damage[11]. We investigated whether enhancement of these markers indicate blood-CSF barriers' dysfunction or due to intrathecal synthesis as a result of the inflammatory process. We also investigated the relationship of these markers to the presence of the organisms. The correlations between S-100B and oxidative stress/antioxidant markers for causal relationship for the severity and neurological complications of bacterial meningitis were discussed.

## Methods

All consecutive children (n = 40; male: 29, female = 11), aged <15 years (mean: 71.8 ± 8.23 months), blood pressure (99 ± 19 mmHg), admitted to the Pediatric Department, Hospital of Infectious Diseases (Fever Hospital), Assiut, Egypt, carried the diagnosis of bacterial meningitis, were included in this study. Septic meningitis was diagnosed by the presence of the following: CSF polymorphonuclear cell count > 5 mm^3^, CSF: blood glucose ratio <60% or CSF glucose < 40 mg/dl and protein > 40 mg/dl in absence of CSF hemorrhage and positive CSF culture[1]. Inclusion was also dependent on the decision to perform a lumbar tap on admission. Twenty healthy children matched for age (mean: 51.5 ± 4.09 months) and sex (male: 12, female = 8) were included for comparison. This study was conducted according to the principles established in Helsinki and approved by Assiut University Hospital ethics committee. Informed consent was obtained from the parents/guardians. Excluded from the study were children with: 1) Sepsis, severe sepsis and septicemic shock[14]. The diagnosis of sepsis was based on the presence of positive blood culture for bacteria in additions to clinical manifestations of sepsis, 2) viral meningitis or encephalitis: Viral CNS infection is diagnosed in the presence of pleocytosis (up to 1000 leukocytes/mm^3^) with predominance of mononuclear cells, normal CSF glucose and chloride concentrations and normal or increased protein. We did not do CSF culture, serology or PCR for neurotropic viruses, 3) fetal and neonatal malformations; chromosomal abnormalities or developmental delay, 4) perinatal asphyxia, 5) immunodeficiency, 6) endocrine diseases as diabetes mellitus and obesity, 7) purpura, 8) impairment of hearing prior to diagnosis of sepsis, 9) neuromuscular diseases, and 10) recent neurosurgery or ventriculoperitoneal shunt.

### Data collection

Clinical data were collected as follow: 1) demographics (age, gender, weight and hospital admission date), coexisting medical conditions, antibiotic pretreatment, vaccination status, temperature and duration of fever at the time of presentation and occurrence and timing of seizures, 2) physical examination findings, 3) routine tests for biochemistry and hematology were done on the date of lumbar tap including complete blood count, kidney and liver function tests, 4) chest x-ray, urine and stool analyses were done as indicated per patient, 5) CSF analysis included white and differential cell count, protein and glucose concentrations, gram-stained CSF smear examination and culture for bacteria, 6) the severity of brain involvement was assessed using the Pediatric Glasgow Coma Scale or scoring system (PGCS)[15]. This is an equivalent of the Glasgow Coma Scale (GCS) used to assess the mental state of adult patients. As many of the assessments for adults would not be appropriate for infants and children below 36 months, the scale was modified slightly. As with the GCS, the PGCS comprises three tests: eye, verbal and motor responses. The three values separately as well as their sum are considered. Accordingly, the degrees of severity are mild (PGCS: 13-15), moderate (PGCS: 9-12) and severe (PGCS: ≤ 8). The lowest possible PGCS (the sum) is 3 (deep coma) whilst the highest is 15 (fully awake and aware), and 7) electroencephalography (EEG) and neuroimaging (CT or MRI brain) were done for all patients.

Upon hospital discharge (survivors or non-survivors), summary data including total hospital stay and complications were collected for all patients.

### Sample Collection

Serum and CSF samples were collected from patients upon admission. Only blood samples were obtained from the control children as our ethical committee did not permit CSF intake from normal children. CSF was collected under strict non-infected conditions. Standard hematological and biochemical analysis of peripheral blood were also documented. All samples were tested for white blood cell count with differential and bacterial cultures. Levels of CSF glucose, protein were determined by routine laboratory procedures. Methods for CSF processing and bacterial culture used in this study have been described previously[16]. Briefly, CSF was inspected for appearance and processed for differential leukocyte cell count and glucose/protein levels. Microbiologic analysis included gram-stain and bacterial culture on blood, MacConkey and chocolate agar plates. Samples collected after hours were inoculated into trans-isolate medium and cultured on plates on the next day. For exclusion of mycobacterium tuberculosis, CSF samples were inoculated into Lowenstein-Jensen medium (Becton Dickinson, Franklin Lakes, NJ, USA). The decision to perform mycobacterium tuberculosis cultures was made at the discretion of the admitting physician. The remaining serum and cerebrospinal fluid samples were spun down, and the supernatant was frozen at -70°C until assayed.

### Mediator assays

#### a) Determination of the serum and CSF concentrations of specific mediators

Nitric oxide (NO) was determined by a classic griess reaction with sulfanilic acid and alpha naphthylamine in diluted sulfuric acid medium as described by Ding et al[17]. LPO was measured by method as described by Grau et al[18]. Superoxide dismutase (SOD) activity and total thiol (total -SH group) content were determined spectrophotometrically according to the method described by Bannister et al[19]. The S-100B protein concentration was measured by a commercially available immunoluminometric assay (ELISA kits, BioVendor Laboratorni Medicina, A.S.) as supplied by the manufacturer's standards. The sensitivity of the kit was 0.1 ng/ml. All serum samples were diluted in diluents buffer provided with the kits while CSF assay was performed on non-diluted samples according to the manufacturer's instructions. Samples and standards were run in duplicate.

#### b) Determination of the CSF-to-serum ratios and indices of specific mediators

Albumin is an exclusively blood-derived CSF protein. It is transferred to the CSF via passive diffusion through the intact blood-CSF barriers. The CSF-to-serum albumin concentration was found to be 1:205. Increased albumin concentrations in CSF must always be due to blood CSF barrier dysfunction. In this study, albumin ratio (albumin CSF concentration × 10^3^/albumin serum concentration) was calculated as an indicator of blood-CSF barrier dysfunction in patients with meningitis[20]. Also by comparing a specific biomarker or mediator ratio (mediator CSF concentration × 10^3^/mediator serum concentration) with the albumin ratio (normalizing to albumin), it is possible to infer whether the mediators of similar molecular weight to albumin are passively transferred across the disrupted blood-CSF barriers or intrathecally synthesized. The latter is indicated by calculation of the blood-derived mediator indices as follow: mediator index is equal to the mediator ratio (CSF/serum) divided by albumin ratio (CSF/serum). The higher the mediator ratio than albumin ratio indicates local intrathecal substance production. Estimation of marker indices seems to have a significance for blood-deriver proteins or substances but it is not applicable for brain-derived proteins or substances as S-100B which has an intrathecal fraction of ~99% (CSF/serum ratio: 18:1; which mean that S-100B is 18 times higher in CSF than the serum)[21]. The serum and CSF albumin were measured by ELISA kit obtained from Egyptian Company for Biotechnology; Cairo, Egypt, detection limit of the assay was 1.0 g/dl.

### Statistical Analysis

The analysis was done using SPSS version 11.5. Data were expressed as mean ± 2SD or as a percentage when appropriate. One-way ANOVA or Mann-Whitney U tests were used for comparison between different groups. The Pearson's and Spearman correlation coefficient tests were used to evaluate associations between measured parameters (continuous and non-continuous variables). For all tests, p < 0.05 was considered significant.

## Results

This is a cross-sectional study. CSF biochemistries of the samples examined confirmed the diagnosis of bacterial meningitis (glucose: 49 ± 26 mg/ml; protein: 94 ± 83 mg/dl; cells count: 58,350 ± 18431 cells/cm^3^, neutrophils: 92 ± 13% and lymphocytes: 8 ± 13%) as compared to our normal lab reference values (glucose: 75 ± 11 mg/ml; protein: 22 ± 7 mg/dl; cells count: ± 4 cells/cm^3^). CSF-culture was positive in 37.5% (n = 15) of patients. Organisms identified were streptococcus pneumonia (n = 6, 40%), staphylococcus aureus (n = 4, 26.7%), haemophilus influenza (n = 3, 20%) and neisseria meningitides (n = 2, 13.3%).

The frequent presenting clinical manifestations include: fever (range: 38-40°C, mean: 39°C), vomiting (n = 27, 67.5%), headache (n = 26, 65%), seizures (n = 22, 55%), nuchal rigidity (n = 17, 42.5%), drowsiness (n = 15, 37.5%), diarrhea (n = 11, 27.5%), cough (n = 10, 25%) and bleeding tendency (n = 1, 2.5%). According to PGCS, moderate (n = 12, 30%) to severe (n = 4, 10%) scores were identified in 40% of children. Fundus examination was normal. Neurological evaluation at the time of hospital discharge (range: 6-13 days, mean: 8.13 ± 1.51) revealed persistence of specific neurological manifestations in 12.5% (n = 5) of patients in the form of squint (6^th ^cranial nerve palsy) (n = 2; 5%) and rigidity of the 4 limbs (n = 3; 7.5%). Headache and drowsiness without other manifestations were observed in 30% (n = 12) which may be non-specific manifestations due to medications. Three patients with rigidity had streptococcus pneumonia and the 2 patients with 6^th ^cranial nerve palsy had haemophilus influenzae and Neisseria meningitidis (one patient each). Mortality rate was 5% (n = 2). Organisms identified were streptococcus pneumonia (n = 6, 40%), staphylococcus aureus (n = 4, 26.7%), haemophilus influenza (n = 3, 20%) and neisseria meningitides (n = 2, 13.3%). The high rate of culture negative disease (n = 25, 62.5%) was caused by multiple factors including the use of antibiotics before admission, a common problem in Egypt and many other developing countries [16] or rapidly processing CSF culture. However, the relatively higher ratio of positive culture (37.5%) despite that 27.5% of patients received antibiotics was attributed to early admission after onset and CSF sampling in the majority of patients.

Table 1 showed the serum and CSF concentrations of albumin, NO, LPO, total thiol, SOD and S-100B protein. Compared to healthy children, serum albumin was lower, while serum S-100B (p < 0.05), NO, LPO, total thiol, SOD were higher in patients with meningitis. Table 2 compared the measured parameters in patients with positive and negative bacterial CSF culture. It was found that serum S-100B, serum NO, total CSF thiol and total thiol index were higher in patients with positive CSF culture compared to patients with negative cultures. In Table 3, S-100B ratio and serum ratio and indices of NO and SOD were higher in patients with neurological complications compared to patients without neurological complications.

**In patients with meningitis**, Positive correlation was found between NO index with CSF white blood cells (r = 0.319, p < 0.05); CSF-LPO with CSF-protein (r = 0.423, p < 0.01); NO and LPO indices (r = 0.318, p < 0.05); total thiol with LPO indices (r = 0.725, p < 0.0001); S-100B and PGCS (0.608, p < 0.0001); CSF-LPO with CSF-S-100B (r = 0.482, p < 0.002); serum-total thiol with serum S-100B (r = 0.423, p < 0.01). In healthy controls, a positive correlation was found between serum S-100B with serum LPO (r = 0.498, p < 0.05).

**Table 1:** Oxidative stress, antioxidant biomarkers and S-100B protein levels in the serum and CSF of the studied group

**Mediators**	**Patients** **(n = 40)**				**Controls** **(n = 20)**
	
	**CSF**	**Serum**	**CSF/serum** **ratio (10^-3^)**	**Index**	**Serum**
Albumin* (g/dl)					
**mean ± 2SD**	1.27 ± 0.08	2.53 ± 0.41	513.78 ± 80.87	-	3.94 ± 1.03
**Range**	1.10-1.51	2.09-3.21	395.64-604.03		2.27-5.40
**Significance**		**p < 0.0001**			

NO (nmol/ml)					
**mean ± 2SD**	16.21 ± 1.73	23.02 ± 7.90	927.34 ± 190.69	1.86 ± 0.54	14.64 ± 2.41
**Range**	13.49-21.55	15.08-45.95	898.03-1453.96	1.25-2.94	10.73-20.62
**Significance**		**p < 0.01**			

LPO (μmol/l)					
**mean ± 2SD**	14.49 ± 1.13	17.89 ± 2.56	825.48 ± 134.26	1.66 ± 0.42	6.78 ± 1.00
**Range**	12.54-15.88	12.38-22.85	642.89-1205.17	1.08-2.50	5.40-8.10
**Significance**		**p < 0.0001**			

Total Thiol (nmol/ml)					
**mean ± 2SD**	0.56 ± 0.20	6.10 ± 2.55	24.11 ± 9.39	0.05 ± 0.02	1.13 ± 0.34
**Range**	0.28-1.25	1.63-10.66	9.06-41.39	0.02-0.1	0.79-1.80
**Significance**		**p < 0.0001**			

SOD (U/g protein)					
**mean ± 2SD**	2.87 ± 1.06	3.30 ± 1.20	122.75 ± 52.70	0.24 ± 0.11	1.08 ± 0.20
**Range**	49.34-2077.48	1.22-6.97	40.43-298.06	0.08-0.62	0.78-1.34
**Significance**		**p < 0.0001**			

S100 B (ng/ml)					
**mean ± 2SD**	0.61 ± 0.05	0.22 ± 0.13	2772.73 ± 188.48	-	0.13 ± 0.02
**Range**	0.55-0.70	0.13-0.57	1228.07-4230.77		0.11-0.16
**Significance**		**p < 0.05**			

One-way ANOVA or *Mann-Whitney U tests were used for comparison between different groups

CSF: cerebrospinal fluid; NO: nitric oxide; LPO: lipid peroxide; SOD: superoxide dismutase

Albumin ratio = [albumin CSF level] × 10^3^

[albumin serum level]

Mediator ratio = [mediator CSF level] × 10^3^

[mediator serum level]

Mediator index = [mediator CSF level]/[mediator serum level] [albumin CSF level]/[albumin serum level]

**Table 2:** Comparison between the serum and CSF concentrations of oxidative stress, antioxidant biomarkers and S-100B protein among patients with positive and negative CSF culture for bacteria

**Mediators**	**Patients with positive bacterial culture (n = 15)**				**Patients with negative bacterial culture (n = 25)**			
	
	**CSF**	**serum**	**CSF/serum** **ratio (10^-3^)**	**Index**	**CSF**	**serum**	**CSF/serum** **ratio (10^-3^)**	**Index**
Albumin* (g/dl)	1.27 ± 0.06	2.50 ± 0.50	526.01 ± 113.21	-	1.29 ± 0.10	2.55 ± 0.43	523.45 ± 109.36	-
**Significance**	p > 0.05	p >0.05	p > 0.05					

NO (nmol/ml)	15.75 ± 1.06	28.55 ± 12.30	694.15 ± 175.22	1.37 ± 0.42	17.24 ± 3.27	23.36 ± 7.77	731.35 ± 194.58	1.44 ± 0.44
**Significance**	p > 0.05	**p < 0.01**	p > 0.05	p > 0.05				

LPO (umol/l)	17.53 ± 3.2	24.31 ± 4.98	741.02 ± 152.27	1.47 ± 0.41	17.79 ± 3.51	24.54 ± 6.41	767.36 ± 217.24	1.53 ± 0.53
**Significance**	p > 0.05	p > 0.05	p > 0.05	p > 0.05				

Total Thiol (nmol/ml)	0.63 ± 0.27	6.09 ± 3.07	26.78 ± 11.35	0.05 ± 0.03	0.52 ± 0.13	6.11 ± 2.25	22.51 ± 7.81	0.04 ± 0.02
**Significance**	**p < 0.01**	p > 0.05	p > 0.05	**p < 0.05**				

SOD (U/g protein)	3.21 ± 1.22	3.89 ± 1.36	133.08 ± 49.53	0.26 ± 0.12	2.66 ± 0.91	2.94 ± 0.94	116.55 ± 54.54	0.23 ± 0.11
**Significance**	p > 0.05	p > 0.05	p > 0.05	p > 0.05				

S100 B (ng/ml)	0.61 ± 0.38	0.70 ± 0.21	945.97 ± 687.62	-	0.66 ± 0.11	0.58 ± 0.42	844.21 ± 555.64	-
**Significance**	p > 0.05	**p < 0.05**	p > 0.05					

One-way ANOVA or *Mann-Whitney U tests were used for comparison between different groups CSF: cerebrospinal fluid; NO: nitric oxide; LPO: lipid peroxide; SOD: superoxide dismutase

**Table 3:** Comparison between the serum and CSF concentrations of oxidative stress, antioxidant biomarkers and S-100B protein among patients with and without neurological complications

**Mediators**	**Patients with neurological complications (n = 7)**				**Patients without neurological complications (n = 35)**			
	
	**CSF**	**serum**	**CSF/serum** **ratio (10^-3^)**	**Index**	**CSF**	**serum**	**CSF/serum** **ratio (10^-3^)**	**Index**
Albumin* (g/dl)	1.35 ± 0.05	2.55 ± 0.43	529.41 ± 94.55	-	1.27 ± 0.43	2.45 ± 0.45	518.37 ± 112.6	-
**Significance**	p > 0.05	p > 0.05	p > 0.05					

NO (nmol/ml)	18.41 ± 1.42	29.70 ± 12.03	620.69 ± 176.07	1.17 ± 0.47	10.91 ± 2.87	22.38 ± 9.68	487.49 ± 190.37	0.94 ± 0.22
**Significance**	**p < 0.05**	**p < 0.05**	**p < 0.05**	**p < 0.05**				

LPO (umol/l)	13.64 ± 2.68	20.24 ± 6.11	673.90 ± 150.47	1.26 ± 0.40	11.70 ± 3.49	28.14 ± 5.84	415.77 ± 200.30	0.80 ± 0.05
**Significance**	**p < 0.05**	**p < 0.05**	**p < 0.01**	**p < 0.05**				

Total Thiol (nmol/ml)	0.69 ± 0.32	6.04 ± 3.07	31.87 ± 12.31	0.07 ± 0.03	0.53 ± 0.16	6.11 ± 2.51	22.74 ± 8.27	0.04 ± 0.02
**Significance**	p > 0.05	p > 0.05	p > 0.05	p > 0.05				

SOD (U/g protein)	3.47 ± 1.30	4.35 ± 1.96	157.47 ± 56.66	0.34 ± 0.11	2.76 ± 0.99	3.11 ± 0.93	116.62 ± 50.37	0.23 ± 0.11
**Significance**	p > 0.05	**p < 0.01**	p > 0.05	p > 0.05				

S100 B (ng/ml)	0.67 ± 0.42	0. 24 ± 0.07	2791.67 ± 754.77	-	0.25 ± 0.40	0.15 ± 0.16	1666.67 ± 573.2	-
**Significance**	**p < 0.01**	**P < 0.01**	**p < 0.001**					

One-way ANOVA or *Mann-Whitney U tests were used for comparison between different groups

CSF: cerebrospinal fluid; NO: nitric oxide; LPO: lipid peroxide; SOD: superoxide dismutase

## Discussion

The results of this study may suggest the following: 1) Dysfunction of blood-CSF barriers occurs with bacterial meningitis, 2) Oxidative stress has an important role in the pathogenesis of bacterial meningitis, 2) Brain damage does occur in bacterial meningitis as evidence by increased intrathecal synthesis of S-100B, NO and LPO biomarkers, 3) the finding of association between the levels of mediators of oxidative stress, antioxidants and S-100B may suggest their role in disease severity and the occurrence of neurological complications.

In the present study, the increased albumin ratio (CSF/serum) is an indicator of morphological and/or biophysical dysfunction of the blood CSF barriers caused by inflammation. Albumin is the main fraction (80%) of CSF proteins. Increased albumin concentrations in CSF must always be due to blood CSF barriers dysfunction (100% blood-derived protein). Furthermore, it has been suggested that the mediators' CSF-to-serum ratio and indices (mediator CSF-to-serum ratio/albumin CSF-to-serum ratio) are better for diagnostic purposes rather than relying on CSF concentrations only and to infer whether the blood-derived mediators of similar molecular weight to albumin are passively transferred across the disrupted blood-CSF barriers or intrathecally synthesized. This has been attributed to the fact the difference in the CSF levels of these mediators throughout the day caused by CSF turnover and the to-and-fro motions of the CSF by cardiac cycles or the circadian rhythms. According to the laws of diffusion, it is important to know that all blood proteins traverse capillary walls of blood-CSF barriers by passive diffusion (molecular flux) into brain, extracellular fluid and CSF according to their molecular weight, e.g. smaller molecules like IgG (150 kDa) (serum/CSF ratio: 500:1), albumin (67 kDa) (serum/CSF ratio: 205:1) are fast in exchange than large molecules e.g. IgM (900 kDa) which are slower in exchange and subsequently form a steeper blood-to-CSF concentration gradient (IgM: serum/CSF ratio: 3400:1)[20,21].

In this study, serum and CSF levels of NO were elevated in children with meningitis. NO CSF/serum ratio and index are suggestive of local production in the CNS as well as passage through the disturbed blood brain barrier. Some authors suggested that the increased level of CSF-NO in patients with bacterial meningitis is due to blood-CSF barriers dysfunction or disturbed permeability as a result of the inflammatory process[22,23]. Bacteria are thought to enter the CNS either by local tissue damage or by transcytosis through microvascular endothelial cells[24,25]. And, as host defense mechanisms are limited in the CSF, bacteria can multiply and reach titers of up to hundred(s) colony-forming units per milliliter. In response to bacterial cell wall components released by autolysis, the rapid increase of proinflammatory cytokines [tumor necrosis factor-a (TNF-a) and interleukins 1b and 6] and chemokines [interleukin-8, macrophage inflammatory protein (MIP)-1 and MIP-2] is followed by the appearance of granulocytes and this resulted in enhanced blood-brain barrier permeability[26]. It has been suggested that NO-related compounds, most probably ONOO- released from infiltrating inflammatory and endothelial cells, may cause loss of integrity of the blood-CSF barriers. Other studies reported no destruction in blood-CSF barriers with meningitis and claimed increased CSF-NO to local production during the course of the inflammatory process which is mediated by cytokines[22]. Marra et al[27]. found infection of the brain without detectable bacteremia in a pneumococcal respiratory tract infection model indicating that pneumococci are able to disseminate by a non-hematogenous route. In contrast, some studies did not find changes in the CSF-NO with bacterial meningitis[28]. A possible explanation for this discrepancy would be a difference in the study subjects and severity of the disease.

A positive correlation was found between NO-index and CSF-WBCs. Another interesting result in this study is that the serum-NO level was higher in patients with gram positive CSF culture compared to negative culture indicating that the NO production is related to the presence of the organisms. Previous reports indicated that higher levels of NO are associated with severe disease [29]. CSF pleocytosis, protein concentration, granulocyte percentage, high tumor necrosis factor-alpha (TNF-α), low glucose and L-arginine during the initial stage of meningitis[22,23]. It has been suggested that beside the bacteria itself, neutrophil accumulation in the subarachnoid space is a hallmark of the acute inflammatory response in bacterial meningitis[30]. Activated neutrophils can secrete large amounts of ROS and a wide variety of potential triggers as cytokines, complement factors and platelet-activating factor in the CSF of patients with meningitis [31,32]. Other possible cell types involved in ROS generation comprise microglia macrophages, cerebral endothelial cells, astrocytes, and neuronal cells[33].

In this study, serum LPO levels were elevated in patients with meningitis which points to the presence of a general status of oxidative stress affecting cellular membranes[5-7,9,26].

In this study, the high levels of NO, regardless of its source, have been associated with membrane lipid peroxidation. There is a substantial body of work implicating ROS, inflammation and oxidative stress in the development of cerebral edema, vascular damage, CSF pleocytosis and intracranial complications in bacterial meningitis and meningoencepahlitis[5,7]. It is known that the brain is a target for free radical damage because of its large lipid content, high quantities of polyunsaturated fatty acids, high rate of metabolism with high consumption of oxygen, and low antioxidant capacity[34]. In the present study, enhancement of antioxidant scavengers' activities were identified as indicated by elevated levels of total thiol and SOD. Serum SOD was higher in patients with neurological complications than those without. In patients with positive bacterial culture, the levels of CSF-total thiol and its index were higher than those with negative culture[5,6,11]. Some studies reported elevation of CSF levels of antioxidant enzymes and malondialdehyde (markers of lipid peroxidation) in children with meningitis[35,36]. In contrast, Aycicek et al[7]. found that serum total oxidant status and lipid hydroperoxide were higher, but CSF total oxidant status and lipid hydroperoxide were lower while CSF total antioxidant status was higher in children with acute bacterial meningitis. Superoxide dismutases (SODs) are metalloenzymes that exists in the form of three isozymes, namely copper-zinc SOD (Cu/Zn SOD), manganese SOD (Mn-SOD) and extracellular SOD, that are widely distributed in human tissues, including those of the nervous system[37]. The medical literature has described SOD as the most important enzyme involved in the removal of superoxide anions. Hirose et al[38]. reported that CSF levels of Mn-SOD were elevated in almost all patients with bacterial meningitis and the mean level of Mn-SOD was higher than those in patients with aseptic meningitis and encephalitis, suggesting that marked elevation of Mn-SOD in the CSF in bacterial meningitis is a phenomenon related to bacterial infection.

In the present study, S-100B concentrations were increased. The results of S-100B CSF/serum ratio suggest increased intrathecal synthesis. Its levels were higher in patients with positive than with negative cultures and in presence of neurological complication than in their absence. A positive correlation was identified between CSF-S-100B and CSF-LPO and between S-100B ratio and the severity of meningitis as indicated by PGCS. The positive correlation was found between serum-S-100B with serum-total thiol lead to the suggestion that the damaged astrocytes may increase the formation of S-100B according to severity of oxidative stress.

S-100B is a low molecular weight mainly brain-derived protein (intrathecal fraction = ~99%; CSF/serum ratio: 18:1). In healthy subjects, natural autoantibodies of IgG class to S-100B are present in serum. However, the exact source of S-100B in the blood is unknown. At normal concentrations, it is able to protect hippocampal neurons against glutamate toxicity[12]. It was already demonstrated that S-100B suppresses oxidative stress induced by cupper[39]. While at higher levels (micromolar), it causes exacerbation of neuroinflammation, oxidative stress and neuronal apoptosis[40]. Increase in S-100B may reflect the extent of glial damage or astrocytic reactions to neural injury (reactive astrogliosis). Gazzolo et al[41]. reported that S-100B was higher in patients with bacterial meningitis without encephalitis and the presence of a correlation between S-100B and CSF pleocytosis could offer additional support to the higher risk of neurological sequelae.

The exact mechanisms that regulate the increased intrathecal levels of S-100B protein in bacterial meningitis are unknown. Inflammation [42]., glutamate [43]. and oxidative stress [44]. are considered as important stimulants for S-100B production.

To summarize, the present investigation provides a new perspective for the clinical study of S-100B, with special reference to neurologic sequelae after bacterial infections. We believe that this study has potential clinical importance in the field of pediatrics and biological processes although the results of this work in some of its parts are confirmatory to the previously established data and our suggestions of their role in the pathogenesis of bacterial meningitis are speculative in nature. This is the main limitation of this study. Further experimental studies are needed to confirm the direct or indirect causal relationship between bacteria, inflammation, oxidative stress and S-100B, markers in the pathogenesis of bacterial meningitis. Although CSF remains the biological fluid of choice, further investigations are needed in other biological fluids.

## Conclusion

The results of this study may have clinical and research implications. It may contribute to further understanding of factors involved in the pathogenesis of brain compromise in bacterial meningitis. It seems to be multifactorial and not due to direct infection of CSF or to the presence of toxins. It involves disruption of the blood-CSF barriers caused by inflammatory process, oxidative stress and impaired astrocyte function. If the results of this study are supported by further research, this may lead to the use of distinct profiles of mediators as possible biomarkers for the prediction of prognosis, monitoring response to treatment and improvement of management strategies of patients with bacterial meningitis.

## Abbreviations

CNS: central nervous system; CSF: cerebrospinal fluid; BBB: blood-brain barrier; LPO: lipid peroxide; NO: nitric oxide; SOD: superoxide dismutase

## Competing interests

The authors declare that they have no competing interests.

## Authors' contributions

SAH carried out the clinical evaluation of the patients, collection of serum and CSF samples from patients, participated in the design of the study, statistical analysis and drafted the manuscript. EAH carried out the laboratory analysis of albumin and S100-B protein from serum and CSF and participated in the design of the study and its statistical analysis. MMZ carried out the laboratory analysis of NO, LPO, total thiol and SOD from serum and CSF and participated in the design of the study. All authors read and approved the final manuscript.

## Pre-publication history

The pre-publication history for this paper can be accessed here:


